# Machine Learning of Schizophrenia Detection with Structural and Functional Neuroimaging

**DOI:** 10.1155/2021/9963824

**Published:** 2021-06-09

**Authors:** Dafa Shi, Yanfei Li, Haoran Zhang, Xiang Yao, Siyuan Wang, Guangsong Wang, Ke Ren

**Affiliations:** Department of Radiology, Xiang'an Hospital of Xiamen University, Xiamen 361002, China

## Abstract

Schizophrenia (SZ) is a severe psychiatric illness, and it affects around 1% of the general population; however, its reliable diagnosis is challenging. Functional MRI (fMRI) and structural MRI (sMRI) are useful techniques for investigating the functional and structural abnormalities of the human brain, and a growing number of studies have reported that multimodal brain data can improve diagnostic accuracy. Machine learning (ML) is widely used in the diagnosis of neuroscience and neuropsychiatry diseases, and it can obtain high accuracy. However, the conventional ML which concatenated the features into a longer feature vector could not be sufficiently effective to combine different features from different modalities. There are considerable controversies over the use of global signal regression (GSR), and few studies have explored the role of GSR in ML in diagnosing neurological diseases. The current study utilized fMRI and sMRI data to implement a new method named multimodal imaging and multilevel characterization with multiclassifier (M3) to classify SZs and healthy controls (HCs) and investigate the influence of GSR in SZ classification. We found that when we used Brainnetome 246 atlas and without performed GSR, our method obtained a classification accuracy of 83.49%, with a sensitivity of 68.69%, a specificity of 93.75%, and an AUC of 0.8491, respectively. We also got great classification performances with different processing methods (with/without GSR and different brain parcellation schemes). We found that the accuracy and specificity of the models without GSR were higher than that of the models with GSR. Our findings indicate that the M3 method is an effective tool to distinguish SZs from HCs, and it can identify discriminative regions to detect SZ to explore the neural mechanisms underlying SZ. The global signal may contain important neuronal information; it can improve the accuracy and specificity of SZ detection.

## 1. Introduction

Schizophrenia (SZ) is a severe psychiatric illness characterized by aberrant sensory perceptions, cognition, concrete thinking, and a restricted range of emotion, and it affects about 1% of the general population [1–5]. SZ is a heterogeneous disorder, and current diagnoses are based on subjective indicators such as self-report, observation, and clinical history, and its reliable diagnosis is challenging [1, 4, 6, 7], and the pathological mechanism is still unclear [4].

Functional MRI (fMRI) and structural MRI (sMRI) are gaining importance and becoming more widely acceptable techniques with the potential to help diagnose neurological illnesses [8–11], including schizophrenia [5, 12, 13]. An increasing number of studies have reported that multimodal brain data can improve diagnostic accuracy by combining the information obtained from different MRI imaging modalities [8, 14–16]. The machine learning (ML) technique is a new approach that can extract relevant information from images and construct models to determine the probability of disease onset, and it can make a higher accurate prediction compared with conventional methods [5, 6, 13, 17, 18]. Salvador et al. [15] achieved 75.76% accuracy in schizophrenia diagnosis, and de Filippis et al. [5] reported that support vector machine associated with other ML techniques could achieve accuracy close to 100%.

In conventional ML methods, most studies usually concatenated the features into a longer feature vector [19–21]. However, these methods may be insufficiently effective to combine different features from different modalities. Some studies [16, 22, 23] have introduced novel methods to convey comprehensive and complementary information more effectively. Dai et al. [16] proposed a new method named multimodal imaging and multilevel characterization with multiclassifier (M3), which can effectively integrate different information from different modalities and can achieve higher classification accuracy than traditional feature combination methods and any single modality feature.

Global signal regression (GSR) is widely used to remove the effects of global BOLD signal variations in the analysis of fMRI studies; however, there are considerable controversies over its implementation [24–26], and few studies have explored the effect of GSR in ML of diagnosing neurological diseases [19, 27]. Different studies have reported inconsistent results on the effect of GSR on SZ [28–30]. As far as we know, very few studies reported the effect of GSR on ML of SZ classification [31]. However, the sample size of that study was limited, and it did not specifically discuss the effect of GSR on SZ classification. The brain parcellation is likely to affect classification accuracy [19, 27]. However, the influence of those factors in SZ classification is not very clear.

In this study, our goals are to classify SZ patients and healthy controls (HCs) using the M3 method and find the most relevant brain regions to explore its potential pathological mechanism. Furthermore, we investigate the influence of GSR and brain parcellation strategy in SZ classification.

## 2. Materials and Methods

### 2.1. Participants

The data in this study were selected from the Center for Biomedical Research Excellence (COBRE) (http://fcon_1000.projects.nitrc.org/indi/retro/cobre.html), an open neuroimaging dataset which includes fMRI and sMRI data from 71 SZs and 74 HCs with complete clinical and imaging information. Diagnostic information was collected by the Structured Clinical Interview used for DSM Disorders (SCID). Subjects were excluded if they had a history of neurological disorder, intellectual disability, severe head trauma with more than 5-minute loss of consciousness, substance abuse, or dependence within the last 12 months. Details of the diagnostic procedure and clinical information are available online (http://fcon_1000.projects.nitrc.org/indi/retro/cobre.html). To rule out the influence of handedness, we only included right-handed participants, so 15 subjects were excluded. And 21 subjects with maximum head motion larger than 2 mm or 2° were removed from the analysis. In the end, 109 subjects (64 HCs and 45 SZs) were included in this study. Demographic information of subjects is summarized in Table 1. Ethical approval was obtained by COBRE investigators; all participants provided written informed consent.

### 2.2. Data Acquisition

All subjects' resting-state fMRI (rs-fMRI) and sMRI data were collected from a 3.0 T Siemens TrioTim scanner. The sMRI data were acquired with T1-weighted magnetization prepared rapid acquisition gradient echo (MPRAGE) sequences: 176 slices, TR = 2530 ms, TE = 1.64, 3.5, 5.36, 7.22, and 9.08 ms, TI = 900 ms, flip angle = 7°, FOV = 256 mm × 256 mm, matrix = 256 × 256, and voxel size = 1 × 1 × 1 mm^3^. Rs-fMRI scans were acquired single-shot echo-planar imaging sequence with the following parameters: 150 volumes, 33 slices, TR = 2000 ms, TE = 29 ms, matrix = 64 × 64, and voxel size = 3.75 × 3.75 × 4.55 mm^3^.

### 2.3. Data Preprocessing, fMRI, and sMRI Index Calculation

All data standard preprocessing was performed by Data Processing & Analysis of Brain Imaging (DPABI, http://rfmri.org/DPABI) [32], which is based on the Statistical Parametric Mapping (SPM12) package (http://www.fil.ion.ucl.ac.uk/spm) and the toolbox for Data Processing Assistant for Resting-State fMRI (DPARSF) toolbox [33] (http://rfmri.org/DPARSF).

The fMRI data preprocessing steps were as follows: (1) The first ten volumes of each subject were removed to ensure a steady-state condition. (2) Slice timing and realignment were carried out, and we excluded the subjects with maximum translation more than 2.0 mm or maximum rotation more than 2.0° in our study. (3) The T1 structural images were segmented into grey matter (GM), white matter (WM), and cerebrospinal fluid (CSF) and were coregistered to the mean functional image by using the Diffeomorphic Anatomical Registration Through Exponentiated Lie Algebra (DARTEL). (4) Functional data were spatially normalized to the Montreal Neurological Institute (MNI) space and resampled to 3 × 3 × 3 mm^3^ voxels. (5) The WM, CSF, 24 head motion parameters, and linear drift were removed as nuisance covariates by a multiple linear regression analysis. (6) Bandpass filter (0.01–0.10 Hz) was used to reduce the effects of low-frequency drift and high-frequency physiological noise.

We calculated the following fMRI measurements: amplitude of low-frequency fluctuations (ALFF) [34, 35], regional homogeneity (ReHo) [35, 36], degree centrality (DC) [37, 38], and voxel-mirrored homotopic connectivity (VMHC) [19, 39]. We used the DPARSF software default settings to calculate these indices. It is worth noting that we did not perform bandpass filter before calculating ALFF, and we set the connection's correlation coefficient threshold of *r* > 0.25 for DC calculation, and the individual functional data was registered to a symmetric template and smoothed with a Gaussian kernel of 4 mm before calculating VMHC. Those functional maps were then performed Fisher-*Z* transformation. Eventually, we smoothed these *Z* maps with a Gaussian kernel of 4 mm except for WMHC.

The GM images obtained from the previous segmentation step were then spatially normalized into standard space, smoothed with a Gaussian kernel of 8 mm, and resampled to 3 × 3 × 3 mm^3^ voxels, and we got grey matter density (GMD) images.

### 2.4. Feature Extraction

**Figure 1:**
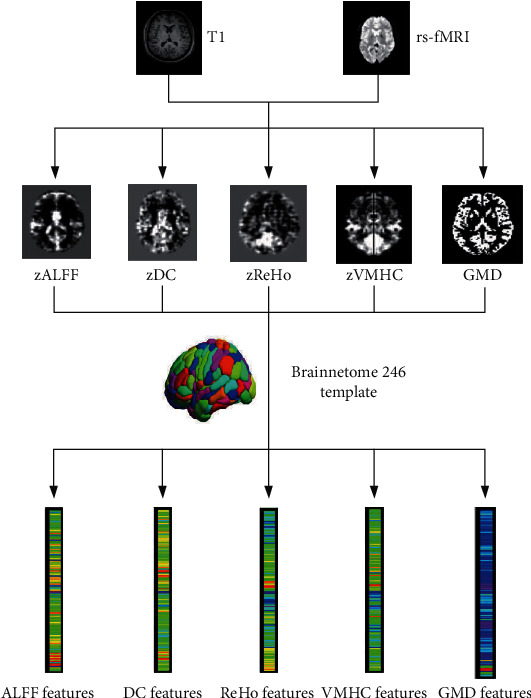
The flowchart of feature extraction. The data preprocessing and index calculation of fMRI and sMRI were performed by the DPABI toolbox, and then, functional maps were then performed Fisher-*Z* transformation. Finally, we obtained fMRI and sMRI measurement maps, including zALFF, zDC, zReHo, zVMHC, and GMD. The fMRI and sMRI maps were segmented into 246 regions of interest using the Brainnetome 246 atlas, and then, we got 246 features for each fMRI and sMRI map. ALFF: amplitude of low-frequency fluctuations; ReHo: regional homogeneity; DC: degree centrality; VMHC: voxel-mirrored homotopic connectivity.

The fMRI and sMRI maps were segmented into 246 regions of interest (ROIs) using the Brainnetome (BN) 246 atlas (see Table S1) [40], which consists of 210 cortical and 36 subcortical subregions in the cerebrum. Each ROI from the brain parcellation atlas was used to mask each individual's fMRI and sMRI map, and the signal value of each ROI was obtained by averaging the fMRI and sMRI signals of all the voxels included in the ROI. This process was repeated for all individuals and regions. Finally, we got 246 features for each fMRI and sMRI map and a total of 1230 features for each individual, as shown in Figure 1. This ROI-based feature extraction method is widely used in neuroimaging ML studies [41–44]. It is an effective method to reduce feature dimensionality and improve computational efficiency [45]. Previous studies have shown that ROI-based feature extraction could denote pathological changes in brain regions, identify abnormal brain regions, and assist in the diagnosis of the disease [20, 41, 46–48]. Then, these features were used in the subsequent analysis.

#### 2.4.1. Discriminative Analysis and Identification of the Most Discriminative Features

**Figure 2:**
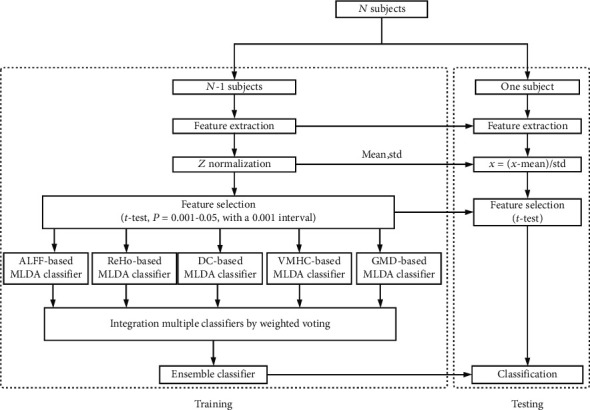
The flowchart of the optimal *P* value threshold selection and M3 method used in our study. We used leave-one-out crossvalidation (LOOCV) to estimate the performance of our classifier. All features in the training and test sets were standardized by *z*-score, and then, two-sample two-tailed *t*-tests with *P* threshold from 0.001 to 0.05 with a 0.001 interval (50 iterations) were performed to select discriminative features. We used MLDA with five category features (ALFF, ReHo, DC, VMHC, and GMD) to obtain five base classifiers, and then, we combined five classifiers into one classifier by weighted voting. Subsequently, we evaluate the performance of the classifier and obtained 50 classification accuracies, and the *P* threshold with the highest classification accuracy was defined as the optimal threshold.

We constructed the model according to the method introduced by Dai et al. [16], which mainly includes feature selection, maximum uncertainty linear discriminate analysis- (MLDA-) based classification, and multiclassifier. Leave-one-out crossvalidation (LOOCV) was conducted to estimate the performance of our classifier (Figure 2).

**Figure 3:**
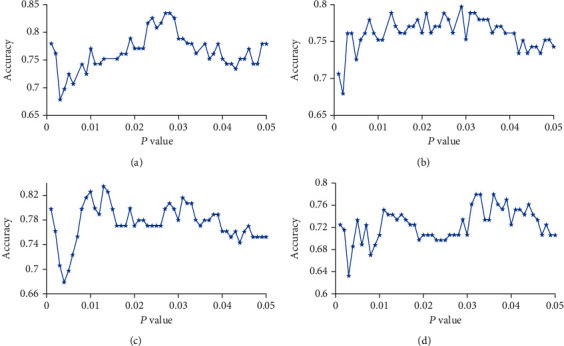
Selection of the optimal *P* threshold via the grid search and LOOCV method. (a–d) Represent the classification accuracy versus the *P* value with BN-246 atlas+noGSR (a), Power-264 atlas+noGSR (b), BN-246 atlas+GSR (c), and Power-264 atlas+GSR (d). BN: Brainnetome atlas; GSR: global signal regression; noGSR: no global signal regression.

The feature selection process was carried out on the training set only. First, all features were standardized by *z*-score, the normalization of the training and test datasets was performed, respectively, and then, two-sample two-tailed *t*-tests were performed to determine the features that showed differences between the SZ patients and HC groups. LOOCV was conducted to estimate the performance of our classifier and to determine the optimal *P* value threshold, as shown in Figures 2 and 3. In brief, we used each subject as the test set to test the performance of the model and the remaining subjects as the training set to train the model, repeated *N* times in turn, where *N* represented the number of subjects. We calculate the performance of the model based on the predicted labels (SZ or HC) and the actual labels of *N* iterations, including accuracy, sensitivity, and specificity. We applied the *P* threshold from 0.001 to 0.05 with a 0.001 interval (50 iterations) and obtained 50 classification accuracies, and the *P* threshold with the highest classification accuracy was defined as the optimal *P* threshold based on the classification accuracy values of the 50 iterations (Figures 2 and 3) [20, 44].

We performed the MLDA-based classifier, multiclassifier, crossvalidation, and identification of the most discriminative feature procedures as previously described [16]. Briefly, we used MLDA with five-category features (ALFF, ReHo, DC, VMHC, and GMD) to obtain five base classifiers. Then, we combined five classifiers into one classifier by weighted voting. Subsequently, LOOCV was used to evaluate the performance of the classifier. For each of the 5 base classifiers, we could obtain the coefficients of the features, and we normalized the coefficients by dividing by the maximum coefficient value. We multiplied the absolute value of normalized coefficients by the base classifier's weight of voting as feature weights. The feature weight of each base classifier is the average of each fold of LOOCV, and finally, we summed the feature weights of each base classifier to obtain the final feature weights of multiclassifier. The most discriminative features were restricted to features that appeared in each fold of LOOCV.

A permutation test was applied 1000 times to test the significance of the prediction performance [20, 44, 47]. First, the class label of each subject was randomly permutated 1000 times without replacement and assigned to all the subjects, and then, the entire M3 procedure was reapplied each time to obtain the permutated classification accuracy, and at last, the *P* value for the accuracy was calculated by dividing the number of permutations that showed a higher accuracy than the actual accuracy for the real sample by the total number of permutations. We applied the same method to calculate the *P* value of AUC by permutating the value of the sum predicted label randomly.

### 2.5. Validating the Influences of GSR and Brain Parcellation

To evaluate the influence of GSR and brain parcellation on our classifier, we did additional analysis: (1) we did regress out global signal in the regressing out nuisance covariate step and (2) we use another additional brain parcellation atlas (Power-264 atlas [49]) to segment brain ROIs. For Power-264 atlas, to be consistent with BN-246 atlas (without cerebellum), we did not include the ROIs of the cerebellum. We performed the same M3 method and evaluated the classification performance. We compare the classification performance of the models with the AUC to determine the effect of GSR and brain parcellation on classifiers with the Delong test.

## 3. Results

### 3.1. Classification Performance

**Figure 4:**
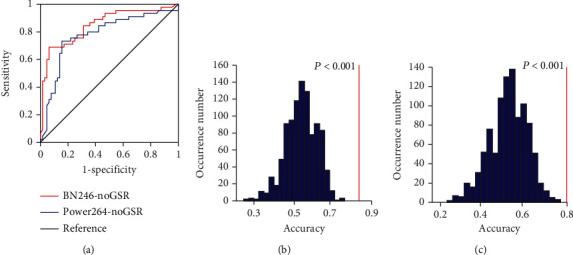
Classification performance of the classifiers without GSR. (a) The ROC curve of the classifiers based on BN-246 atlas and Power-264 atlas without GSR. The distributions of the permutated accuracy values of BN-246 atlas (b) and Power-264 atlas (c) without GSR. The red line indicates the values obtained using the real labels. BN: Brainnetome atlas; noGSR: no global signal regression.

To optimize the *P* threshold value to select the features of the classifier, we performed the grid search method using the training set. We applied the *P* threshold from 0.001 to 0.05 with a 0.001 interval, we found that the optimal *P* threshold was 0.027, and the corresponding classifier obtained a classification accuracy of 83.49%, with a sensitivity of 68.69%, a specificity of 93.75%, and an AUC of 0.8491, respectively (Table 2, Figures 3(a) and 4(a)). The *P* values of the model accuracy and AUC both were *P* < 0.001 (Figure 4(b), Figure S1a), which suggests that the classifier prediction performance was significantly higher than chance.

### 3.2. Discriminative Brain Regions

**Figure 5:**
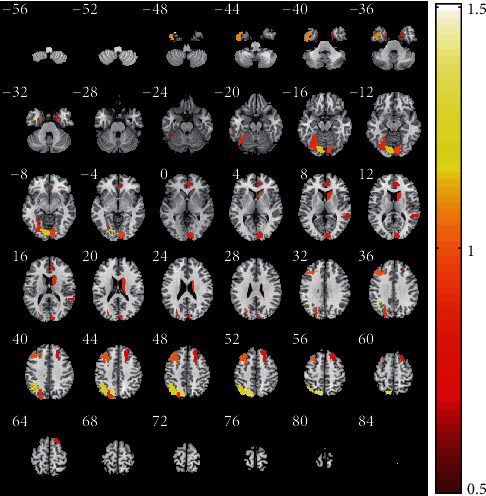
The most discriminative brain regions. The most discriminative regions included the left superior parietal lobule, inferior parietal lobule, inferior temporal gyrus, middle frontal gyrus, lateral occipital cortex, fusiform gyrus, right basal ganglia, cingulate gyrus, superior frontal gyrus, posterior superior temporal sulcus, bilateral medioventral occipital cortex, and parahippocampal gyrus. The color bar value represents the weight value of the brain regions.

To determine which brain regions contributed to single-subject classification, we computed the model feature weights and obtained the order of feature contribution of the classification [16]. The most discriminative features were restricted to features that appeared in each fold of LOOCV. The top 15 brain regions with the highest feature weights are reported in Table 3 and Figure 5. The most discriminative regions included the left superior parietal lobule, inferior parietal lobule, inferior temporal gyrus, middle frontal gyrus, lateral occipital cortex, fusiform gyrus, right basal ganglia, cingulate gyrus, superior frontal gyrus, posterior superior temporal sulcus, bilateral medioventral occipital cortex, and parahippocampal gyrus (Table 3; Figure 5).

### 3.3. Influence of Brain Regional Parcellation Schemes

To evaluate the influence of the parcellation schemes on our M3 classifier, we performed our analysis approach with Power-264 atlas to test the classification performance of the model. We obtained a high classification performance with AUC of 0.7785, and the accuracy, sensitivity, and specificity were 79.82%, 73.33%, and 84.38%, respectively (Table 2, Figures 3(b), 4(a), and 4(c), Figure S1b). We compared the AUC of two classifier performances using the Delong test, and we did not find a significant difference between them (*z* = 0.095, *P* = 0.92).

### 3.4. Influence of Global Signal Regression

**Figure 6:**
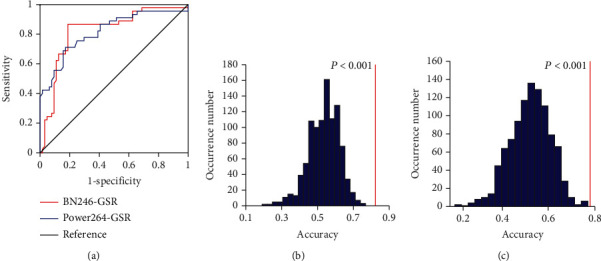
Classification performance of the classifiers with GSR. (a) The ROC curve of the classifiers based on BN-246 atlas and Power-264 atlas with GSR. The distributions of the permutated accuracy values of BN-246 atlas (b) and Power-264 atlas (c) with GSR. The red line indicates the values obtained using the real labels. BN: Brainnetome atlas; AAL: automated anatomical labeling; GSR: global signal regression.

In order to evaluate the influence of global signal regression, we performed the above M3 methods with GSR. We found that the accuracy and specificity of the classifiers without GSR were equal or higher than that of the classifiers with GSR both with BN-246 atlas and Power-264 atlas (Table 2; Figures 3(c), 3(d), and 6 and Figure S2), but we did not find significant differences between them (BN-246_GSR vs. BN-246_noGSR: *z* = −0.74, *P* = 0.46; Power-264_GSR vs. Power-264_noGSR: *z* = 0.93, *P* = 0.35).

## 4. Discussion

In the current study, we combined functional and structural MRI measures to distinguish SZs from HCs by the M3 method. We found that we got great classification performances using different processing methods (with/without GSR and different brain regional parcellation schemes) and a set of discriminative regions to distinguish SZs from HCs. Our study demonstrates that the M3 method is a great tool to effectively distinguish SZs from HCs and explore the neural mechanisms underlying SZ.

Previous neuroimaging ML studies focused on a single modal image [50, 51] or concatenated multimodal features into a longer feature vector [19, 21, 44, 47]. Recent studies have shown that multimodal imaging using integrated information can significantly improve the classification accuracy [16, 20, 21, 23, 47]. And conventional multimodal direct feature concatenation method may not be sufficiently effective in combining features from different modalities [16, 52]. Previous studies have shown that the M3 method can improve classification performance than any single-modal feature and conventional multimodal feature combination methods [16]. Our study also found that the M3 method can effectively distinguish SZs from HCs. Dai et al. [16] found that the M3 method can identify the most discriminative features to classify Alzheimer's disease patients and HCs which are consistent with previous studies that have used conventional univariate statistical analysis of structural and functional MRI. It may be able to explore the underlying mechanisms of neuropsychiatric diseases. Our results are consistent with the previous study.

In previous studies, most researchers empirically chose *P* < 0.01 or *P* < 0.05 as the threshold for feature selection in the data dimensionality reduction step [16, 18, 19]. In our study, we performed grid search [20, 44, 53] to select the optimal *P* threshold to obtain the relatively highest prediction accuracy of the classifier. Most current ML studies [8, 19, 21, 47] usually concatenated the features into a longer feature vector. However, these methods are insufficiently effective to combine different features from different modalities. It has been confirmed that the M3 method can effectively integrate different information from different modalities and can achieve higher classification accuracy than traditional feature combination methods and any single modality features [16]. In our study, we used the BN-246 atlas and M3 method with five types of modality features (ALFF, ReHo, DC, VMHC, and GMD), and we obtained a classification accuracy of 83.49%, a sensitivity of 68.69%, and a specificity of 93.75%, respectively. In addition, we obtained close classification performance using different preprocessing methods (with/without GSR) and different brain regional parcellation schemes. Our classification accuracy is close to or even higher than the results of SZ ML studies [1, 2, 5, 6, 14, 18] and other disease studies [9, 19, 44, 51]. These results are consistent with a previous study which reported that the M3 method can obtain great classification accuracy [16].

The debate about the complex composition of global signals and the necessity of GSR in data preprocessing and data analysis always exists in most cases [24, 54, 55]. Some studies reported that the global signal was likely to reflect important neuronal components in rs-fMRI data [17, 18, 39]. Qing et al. [26] reported that GSR effects are region-specific and suggested that it is great to report results both with and without GSR in ReHo study. Li et al. [56] reported that GSR strengthens association between resting-state functional connectivity and behavior in young healthy adults. And in some studies [57, 58], the authors regressed out global signal as a nuisance variable to reduce the effects of nonneuronal BOLD fluctuations.

Controversies also root in the influence of the global signal in distinguishing SZs from HCs. Yang et al. [28] reported that the variance of the global BOLD signal was significantly higher in patients with SZ as compared to HCs, but not in bipolar disorder, they pointed that the global signal had important biological significance for SZ, and its effects were disease-specific. Similarly, Hahamy et al. [29] reported that GSR reduced variance in subjects with SZ. On the contrary, a recent study showed that there was no overall increase or reduction in resting-state global signal in SZ patients [30]. In our study, we did not find a significant difference in classification performance between the classifiers with and without GSR, which is consistent with a previous study [19]. These results indicated that our model was robust, but we found that the accuracy and specificity of the models without GSR were higher than that of the models with GSR both with BN-246 atlas and Power-264 atlas (Table 2, Figures 4 and 6), which are consistent with previous studies [27, 28]. This could facilitate accurate SZ patient identification and early intervention. Chen et al. reported [27] that the model without GSR enhanced the sensitivity of the detection of differences between Alzheimer's disease and HCs. A recent study also found that global network metrics without GSR have more significant differences than that with GSR between major depression patients and HCs. For SZ, previous studies reported that the global signal was of functional relevance, as it differentiated between SZs and HCs [28, 29], which are consistent with our result, but Umeh et al. [30] reported a contradictory finding. However, in that study, the sample size was limited (38 SZs and 35 HCs). All these findings suggest that the global signal may contain important neuronal information, at least in the case of SZ.

We repeated our M3 pipeline using Power-264 atlas to evaluate whether our results were affected by brain parcellation. Previous studies reported that functionally defined parcellation or high spatial resolution parcellation is probably to detect more significant differences, and anatomical boundaries do not necessarily match functional ones [27, 59]. Therefore, we utilized functional atlas in our study (BN-246 and Power-264 atlas). In our study, we got high classification performances and did not find any significant difference in different brain regional parcellation schemes, which suggested that brain parcellation did not influence our prediction performance, and it is consistent with previous studies [19, 60]. The result indicated that our model had good robustness and generalizability.

Many studies reported abnormal structures and functional brain regions of SZ. In the current study, we found that the most discriminate regions between SZs and HCs mainly locate in the left superior parietal lobule, inferior parietal lobule, inferior temporal gyrus, middle frontal gyrus, lateral occipital cortex, fusiform gyrus, right basal ganglia, cingulate gyrus, superior frontal gyrus, posterior superior temporal sulcus, bilateral medioventral occipital cortex, and parahippocampal gyrus (Figure 5). A meta-analysis that included 79 studies reported that SZ was associated with structural and functional abnormalities in the bilateral anterior cingulate gyrus and middle frontal gyrus [61]. Ren et al. [62] also reported the abnormalities in bilateral anterior cingulate gyrus cortex, occipital gyrus, and left inferior parietal lobule in drug-naive first-episode SZ patients. Zhao et al. [63] reported that SZ patients had extensive structural and functional abnormalities, including bilateral occipital lobe, left orbital frontal cortex, bilateral superior parietal lobule, right middle temporal gyrus, gyrus rectus and superior frontal gyrus, bilateral inferior parietal lobule, and precuneus. Machine learning studies also reported that the bilateral fusiform gyrus, superior parietal lobule, superior temporal gyrus, cingulate gyrus, middle frontal gyrus, inferior parietal lobule, parahippocampal gyrus, and right medial superior frontal gyrus contributed to discrimination between SZ patients and HCs [3, 5, 7, 18, 64–66]. Our results are consistent with these previous studies.

Our study has some limitations. First, although the sample size is limited, it is relatively larger when compared to previous studies [1, 21, 31, 50, 65]. A larger sample size should be recruited in the future to replicate and enrich our findings. Second, some studies [5, 67] have reported differences in the cerebellum between SZ patients and HCs, but in some cases, in our study, the cerebellum was not fully covered during rs-fMRI scanning, so the cerebellum was not considered in our study, and we selected the atlas that does not contain the cerebellum or excluded the cerebellar ROI in the brain atlas. Third, the head motion in subjects with SZ was greater than that in subjects with HC (mean framewise displacement Jenkinson: SZ, 0.19 ± 0.10 mm; HC, 0.14 ± 0.08 mm). Although we adopted strict control inclusion criteria (less than 2 mm and 2.0°), regressed out head motion (with 24 head motion parameters), we cannot guarantee the complete removal of the head motion effect.

## 5. Conclusion

In conclusion, our findings indicated that the M3 method is a great tool to distinguish SZs from HCs effectively with high classification accuracy; it can be generalized in different brain parcellation schemes. The global signal may contain important neuronal information; it can improve the accuracy and specificity to detect SZ patients. The M3 method without GSR is helpful to identify patient accuracy and early intervention, and it can identify discriminative regions to detect SZ to explore the neural mechanisms underlying SZ.

## Figures and Tables

**Table 1:** Demographic and clinical data.

Items	SZs	HCs	Statistic	*P* value
Sample size	45	64	—	—
Gender (M/F)	36/9	44/20	1.71^a^	0.19
Age (years)	37.42 ± 13.55	35.22 ± 11.40	0.92^b^	0.26

^a^The chi-squared value was obtained by the chi-square test. ^b^The *t* value was obtained by the two-sample two-tailed *t*-test. SZ: schizophrenia; HC: healthy control.

**Table 2:** Classification performance of the M3 classifiers.

Items	Optimal *P* threshold	AUC	Accuracy (%)	Sensitivity (%)	Specificity (%)
BN-246_noGSR	0.027	0.8491	83.49	68.89	93.75
Power-264_noGSR	0.029	0.7785	79.82	73.33	84.38
BN-246_GSR	0.013	0.8215	83.49	86.67	81.25
Power-264_GSR	0.032	0.8165	77.98	71.11	82.81

M3: multimodal imaging and multilevel characterization with multiclassifier; BN: Brainnetome atlas; GSR: global signal regression; noGSR: no global signal regression.

**Table 3:** Top 15 most discriminative features for classification.

Regions	ALFF	DC	ReHo	VMHC	GMD	Weight
SPL_L_5_2	*t* = −3.22 (*P* = 0.0017)	*t* = −2.69 (*P* = 0.0082)	NS	*t* = −2.62 (*P* = 0.0102)	NS	1.3275
IPL_L_6_2	NS	*t* = 2.70 (*P* = 0.0080)	*t* = 2.97 (*P* = 0.0037)	NS	NS	1.2037
MVOcC_L_5_1	*t* = −2.77 (*P* = 0.0066)	*t* = −4.49 (*P* < 0.0001)	*t* = −3.75 (*P* = 0.0002)	*t* = −3.82 (*P* = 0.0002)	NS	1.1467
ITG_L_7_3	*t* = 4.78 (*P* < 0.0001)	*t* = 4.18 (*P* < 0.0001)	*t* = 3.66 (*P* = 0.0004)	NS	NS	1.0778
MFG_L_7_5	*t* = 2.66 (*P* = 0.0091)	*t* = 3.54 (*P* = 0.0006)	*t* = 2.83 (*P* = 0.0056)	*t* = 2.88 (*P* = 0.0048)	*t* = −2.42 (*P* = 0.0172)	0.9969
MVOcC _R_5_1	NS	*t* = −2.85 (*P* = 0.0053)	*t* = −3.94 (*P* = 0.0001)	*t* = −3.44 (*P* = 0.0008)	NS	0.9484
PhG_L_6_5	NS	*t* = 3.10 (*P* = 0.0025)	NS	*t* = 2.86 (*P* = 0.0051)	*t* = −2.96 (*P* = 0.0037)	0.9476
LOcC_L_2_2	*t* = −3.18 (*P* = 0.0019)	*t* = −3.57 (*P* = 0.0005)	*t* = −2.69 (*P* = 0.0084)	*t* = −4.00 (*P* = 0.0001)	NS	0.9099
FuG_L_3_2	*t* = −2.81 (*P* = 0.0058)	*t* = −4.55 (*P* < 0.0001)	*t* = −3.49 (*P* = 0.0007)	*t* = −3.56 (*P* = 0.0006)	NS	0.8904
BG_R_6_5	NS	*t* = 2.70 (*P* = 0.0081)	*t* = 3.40 (*P* = 0.0010)	*t* = 2.39 (*P* = 0.0188)	*t* = −3.84 (*P* = 0.0002)	0.8877
MVOcC_R_5_3	*t* = −2.46 (*P* = 0.016)	*t* = −2.89 (*P* = 0.0046)	*t* = −3.12 (*P* = 0.0023)	*t* = −3.75 (*P* = 0.0003)	NS	0.8413
CG_R_7_7	NS	NS	*t* = −3.07 (*P* = 0.0027)	*t* = −2.35 (*P* = 0.0204)	NS	0.7860
SFG_R_7_2	*t* = 3.89 (*P* = 0.0002)	*t* = 3.45 (*P* = 0.0008)	*t* = 3.95 (*P* = 0.0001)	*t* = 2.60 (*P* = 0.0107)	NS	0.7672
PhG_R_6_5	NS	*t* = 3.59 (*P* = 0.0005)	NS	*t* = 2.61 (*P* = 0.0104)	NS	0.7664
pSTS_R_2_2	NS	*t* = −4.05 (*P* < 0.0001)	*t* = −2.28 (*P* = 0.0246)	*t* = −2.76 (*P* = 0.0068)	*t* = −2.78 (*P* = 0.0064)	0.7283

NS: not significant, *P* > 0.027 (optimal *P* threshold); positive *t* value means increased values in the SZ group. ALFF: amplitude of low-frequency fluctuations (ALFF); ReHo: regional homogeneity; DC: degree centrality; VMHC: voxel-mirrored homotopic connectivity; SPL: superior parietal lobule; IPL: inferior parietal lobule; MVOcC: medioventral occipital cortex; ITG: inferior temporal gyrus; MFG: middle frontal gyrus; PhG: parahippocampal gyrus; LOcC: lateral occipital cortex; FuG: fusiform gyrus; BG: basal ganglia; CG: cingulate gyrus; SFG: superior frontal gyrus; pSTS: posterior superior temporal sulcus; L: left; R: right.

## Data Availability

Publicly available datasets were analyzed in this study. This data can be found here: http://fcon_1000.projects.nitrc.org/indi/retro/cobre.html. The code is available on request to the corresponding author.

## Supplementary Materials

Supplementary Materials Table S1: cortical and subcortical regions of interest defined in Brainnetome atlas. Figure S1: the distributions of the permutated AUC values without GSR. The distributions of the permutated AUC values of BN-246 atlas (a) and Power-264 atlas (b) without GSR. The red line indicates the values obtained using the real sum predicted label. Figure S2: the distributions of the permutated AUC values with GSR. The distributions of the permutated AUC values of BN-246 atlas (a) and Power-264 atlas (c) with GSR. The red line indicates the values obtained using the real sum predicted label.
