# Supplementary material for: Machine Learning of Schizophrenia Detection with Structural and Functional Neuroimaging
Source: Dis Markers. 2021 Jun 9;2021:9963824. doi: 10.1155/2021/9963824 (PMC8208855; doi:10.1155/2021/9963824)
Supplement: Supplementary Materials — Table S1: cortical and subcortical regions of interest defined in Brainnetome atlas. Figure S1: the distributions of the permutated AUC values without GSR. The distributions of the permutated AUC values of BN-246 atlas (a) and Power-264 atlas (b) without GSR. The red line indicates the values obtained using the real sum predicted label. Figure S2: the distributions of the permutated AUC values with GSR. The distributions of the permutated AUC values of BN-246 atlas (a) and Power-264 atlas (c) with GSR. The red line indicates the values obtained using the real sum predicted label. [file 9963824.f1.DOCX]

Supplementary Material

Table S1. Cortical and Subcortical Regions of Interest Defined in Brainnetome atlas

| **Lobe** | **Gyrus** | **Regions** | **Index** | **Anatomical and modified Cyto-architectonic descriptions** |
| --- | --- | --- | --- | --- |
| **Frontal Lobe** | SFG (Superior Frontal Gyrus) | SFG_L(R)_7_1 | 1,2 | *A8m, medial area 8* |
|  |  | SFG_L(R)_7_2 | 3,4 | *A8dl, dorsolateral area 8* |
|  |  | SFG_L(R)_7_3 | 5,6 | *A9l, lateral area 9* |
|  |  | SFG_L(R)_7_4 | 7,8 | *A6dl, dorsolateral area 6* |
|  |  | SFG_L(R)_7_5 | 9,10 | *A6m, medial area 6* |
|  |  | SFG_L(R)_7_6 | 11,12 | *A9m,medial area 9* |
|  |  | SFG_L(R)_7_7 | 13,14 | *A10m, medial area 10* |
|  | MFG (Middle Frontal Gyrus) | MFG_L(R)_7_1 | 15,16 | *A9/46d, dorsal area 9/46* |
|  |  | MFG_L(R)_7_2 | 17,18 | *IFJ, inferior frontal junction* |
|  |  | MFG_L(R)_7_3 | 19,20 | *A46, area 46* |
|  |  | MFG_L(R)_7_4 | 21,22 | *A9/46v, ventral area 9/46* |
|  |  | MFG_L(R)_7_5 | 23,24 | *A8vl, ventrolateral area 8* |
|  |  | MFG_L(R)_7_6 | 25,26 | *A6vl, ventrolateral area 6* |
|  |  | MFG_L(R)_7_7 | 27,28 | *A10l, lateral area10* |
|  | IFG (Inferior Frontal Gyrus) | IFG_L(R)_6_1 | 29,30 | *A44d,dorsal area 44* |
|  |  | IFG_L(R)_6_2 | 31,32 | *IFS, inferior frontal sulcus* |
|  |  | IFG_L(R)_6_3 | 33,34 | *A45c, caudal area 45* |
|  |  | IFG_L(R)_6_4 | 35,36 | *A45r, rostral area 45* |
|  |  | IFG_L(R)_6_5 | 37,38 | *A44op, opercular area 44* |
|  |  | IFG_L(R)_6_6 | 39,40 | *A44v, ventral area 44* |
|  | OrG (Orbital Gyrus) | OrG_L(R)_6_1 | 41,42 | *A14m, medial area 14* |
|  |  | OrG_L(R)_6_2 | 43,44 | *A12/47o, orbital area 12/47* |
|  |  | OrG_L(R)_6_3 | 45,46 | *A11l, lateral area 11* |
|  |  | OrG_L(R)_6_4 | 47,48 | *A11m, medial area 11* |
|  |  | OrG_L(R)_6_5 | 49,50 | *A13, area 13* |
|  |  | OrG_L(R)_6_6 | 51,52 | *A12/47l, lateral area 12/47* |
|  | PrG (Precentral Gyrus) | PrG_L(R)_6_1 | 53,54 | *A4hf, area 4(head and face region)* |
|  |  | PrG_L(R)_6_2 | 55,56 | *A6cdl, caudal dorsolateral area 6* |
|  |  | PrG_L(R)_6_3 | 57,58 | *A4ul, area 4(upper limb region)* |
|  |  | PrG_L(R)_6_4 | 59,60 | *A4t, area 4(trunk region)* |
|  |  | PrG_L(R)_6_5 | 61,62 | *A4tl, area 4(tongue and larynx region)* |
|  |  | PrG_L(R)_6_6 | 63,64 | *A6cvl, caudal ventrolateral area 6* |
|  | PCL (Paracentral Lobule) | PCL_L(R)_2_1 | 65,66 | *A1/2/3ll, area1/2/3 (lower limb region)* |
|  |  | PCL_L(R)_2_2 | 67,68 | *A4ll, area 4, (lower limb region)* |
| **Temporal Lobe** | STG (Superior Temporal Gyrus) | STG_L(R)_6_1 | 69,70 | *A38m, medial area 38* |
|  |  | STG_L(R)_6_2 | 71,72 | *A41/42, area 41/42* |
|  |  | STG_L(R)_6_3 | 73,74 | *TE1.0 and TE1.2* |
|  |  | STG_L(R)_6_4 | 75,76 | *A22c, caudal area 22* |
|  |  | STG_L(R)_6_5 | 77,78 | *A38l, lateral area 38* |
|  |  | STG_L(R)_6_6 | 79,80 | *A22r, rostral area 22* |
|  | MTG (Middle Temporal Gyrus) | MTG_L(R)_4_1 | 81,82 | *A21c, caudal area 21* |
|  |  | MTG_L(R)_4_2 | 83,84 | *A21r, rostral area 21* |
|  |  | MTG_L(R)_4_3 | 85,86 | *A37dl, dorsolateral area37* |
|  |  | MTG_L(R)_4_4 | 87,88 | *aSTS, anterior superior temporal sulcus* |
|  | ITG (Inferior Temporal Gyrus) | ITG_L(R)_7_1 | 89,90 | *A20iv, intermediate ventral area 20* |
|  |  | ITG_L(R)_7_2 | 91,92 | *A37elv, extreme lateroventral area37* |
|  |  | ITG_L(R)_7_3 | 93,94 | *A20r, rostral area 20* |
|  |  | ITG_L(R)_7_4 | 95,96 | *A20il, intermediate lateral area 20* |
|  |  | ITG_L(R)_7_5 | 97,98 | *A37vl, ventrolateral area 37* |
|  |  | ITG_L(R)_7_6 | 99,100 | *A20cl, caudolateral of area 20* |
|  |  | ITG_L(R)_7_7 | 101,102 | *A20cv, caudoventral of area 20* |
|  | FuG (Fusiform Gyrus) | FuG_L(R)_3_1 | 103,104 | *A20rv, rostroventral area 20* |
|  |  | FuG_L(R)_3_2 | 105,106 | *A37mv, medioventral area37* |
|  |  | FuG_L(R)_3_3 | 107,108 | *A37lv, lateroventral area37* |
|  | PhG (Parahippocampal Gyrus) | PhG_L(R)_6_1 | 109,110 | *A35/36r, rostral area 35/36* |
|  |  | PhG_L(R)_6_2 | 111,112 | *A35/36c, caudal area 35/36* |
|  |  | PhG_L(R)_6_3 | 113,114 | *TL, area TL (lateral PPHC, posterior parahippocampal gyrus)* |
|  |  | PhG_L(R)_6_4 | 115,116 | *A28/34, area 28/34 (EC, entorhinal cortex)* |
|  |  | PhG_L(R)_6_5 | 117,118 | *TI, area TI(temporal agranular insular cortex)* |
|  |  | PhG_L(R)_6_6 | 119,120 | *TH, area TH (medial PPHC)* |
|  | pSTS (posterior Superior Temporal Sulcus) | pSTS_L(R)_2_1 | 121,122 | *rpSTS, rostroposterior superior temporal sulcus* |
|  |  | pSTS_L(R)_2_2 | 123,124 | *cpSTS, caudoposterior superior temporal sulcus* |
| **Parietal Lobe** | SPL (superior Parietal Lobule) | SPL_L(R)_5_1 | 125,126 | *A7r, rostral area 7* |
|  |  | SPL_L(R)_5_2 | 127,128 | *A7c, caudal area 7* |
|  |  | SPL_L(R)_5_3 | 129,130 | *A5l, lateral area 5* |
|  |  | SPL_L(R)_5_4 | 131,132 | *A7pc, postcentral area 7* |
|  |  | SPL_L(R)_5_5 | 133,134 | *A7ip, intraparietal area 7(hIP3)* |
|  | IPL (Inferior Parietal Lobule) | IPL_L(R)_6_1 | 135,136 | *A39c, caudal area 39(PGp)* |
|  |  | IPL_L(R)_6_2 | 137,138 | *A39rd, rostrodorsal area 39(Hip3)* |
|  |  | IPL_L(R)_6_3 | 139,140 | *A40rd, rostrodorsal area 40(PFt)* |
|  |  | IPL_L(R)_6_4 | 141,142 | *A40c, caudal area 40(PFm)* |
|  |  | IPL_L(R)_6_5 | 143,144 | *A39rv, rostroventral area 39(PGa)* |
|  |  | IPL_L(R)_6_6 | 145,146 | *A40rv, rostroventral area 40(PFop)* |
|  | Pcun (Precuneus) | PCun_L(R)_4_1 | 147,148 | *A7m, medial area 7(PEp)* |
|  |  | PCun_L(R)_4_2 | 149,150 | *A5m, medial area 5(PEm)* |
|  |  | PCun_L(R)_4_3 | 151,152 | *dmPOS, dorsomedial parietooccipital sulcus(PEr)* |
|  |  | PCun_L(R)_4_4 | 153,154 | *A31, area 31 (Lc1)* |
|  | PoG (Postcentral Gyrus) | PoG_L(R)_4_1 | 155,156 | *A1/2/3ulhf, area 1/2/3(upper limb, head and face region)* |
|  |  | PoG_L(R)_4_2 | 157,158 | *A1/2/3tonIa, area 1/2/3(tongue and larynx region)* |
|  |  | PoG_L(R)_4_3 | 159,160 | *A2, area 2* |
|  |  | PoG_L(R)_4_4 | 161,162 | *A1/2/3tru, area1/2/3(trunk region)* |
| **Insular Lobe** | INS (Insular Gyrus) | INS_L(R)_6_1 | 163,164 | *G, hypergranular insula* |
|  |  | INS_L(R)_6_2 | 165,166 | *vIa, ventral agranular insula* |
|  |  | INS_L(R)_6_3 | 167,168 | *dIa, dorsal agranular insula* |
|  |  | INS_L(R)_6_4 | 169,170 | *vId/vIg, ventral dysgranular and granular insula* |
|  |  | INS_L(R)_6_5 | 171,172 | *dIg, dorsal granular insula* |
|  |  | INS_L(R)_6_6 | 173,174 | *dId, dorsal dysgranular insula* |
| **Limbic Lobe** | CG (Cingulate Gyrus) | CG_L(R)_7_1 | 175,176 | *A23d, dorsal area 23* |
|  |  | CG_L(R)_7_2 | 177,178 | *A24rv, rostroventral area 24* |
|  |  | CG_L(R)_7_3 | 179,180 | *A32p, pregenual area 32* |
|  |  | CG_L(R)_7_4 | 181,182 | *A23v, ventral area 23* |
|  |  | CG_L(R)_7_5 | 183,184 | *A24cd, caudodorsal area 24* |
|  |  | CG_L(R)_7_6 | 185,186 | *A23c, caudal area 23* |
|  |  | CG_L(R)_7_7 | 187,188 | *A32sg, subgenual area 32* |
| **Occipital Lobe** | MVOcC (MedioVentral Occipital Cortex) | MVOcC _L(R)_5_1 | 189,190 | *cLinG, caudal lingual gyrus* |
|  |  | MVOcC _L(R)_5_2 | 191,192 | *rCunG, rostral cuneus gyrus* |
|  |  | MVOcC _L(R)_5_3 | 193,194 | *cCunG, caudal cuneus gyrus* |
|  |  | MVOcC _L(R)_5_4 | 195,196 | *rLinG, rostral lingual gyrus* |
|  |  | MVOcC _L(R)_5_5 | 197,198 | *vmPOS,ventromedial parietooccipital sulcus* |
|  | LOcC (lateral Occipital Cortex) | LOcC_L(R)_4_1 | 199,200 | *mOccG, middle occipital gyrus* |
|  |  | LOcC _L(R)_4_2 | 201,202 | *V5/MT+, area V5/MT+* |
|  |  | LOcC _L(R)_4_3 | 203,204 | *OPC, occipital polar cortex* |
|  |  | LOcC_L(R)_4_4 | 205,206 | *iOccG, inferior occipital gyrus* |
|  |  | LOcC _L(R)_2_1 | 207,208 | *msOccG, medial superior occipital gyrus* |
|  |  | LOcC _L(R)_2_2 | 209,210 | *lsOccG, lateral superior occipital gyrus* |
| **Subcortical Nuclei** | Amyg (Amygdala) | Amyg_L(R)_2_1 | 211,212 | *mAmyg, medial amygdala* |
|  |  | Amyg_L(R)_2_2 | 213,214 | *lAmyg, lateral amygdala* |
|  | Hipp (Hippocampus) | Hipp_L(R)_2_1 | 215,216 | *rHipp, rostral hippocampus* |
|  |  | Hipp_L(R)_2_2 | 217,218 | *cHipp, caudal hippocampus* |
|  | BG (Basal Ganglia) | BG_L(R)_6_1 | 219,220 | *vCa, ventral caudate* |
|  |  | BG_L(R)_6_2 | 221,222 | *GP, globus pallidus* |
|  |  | BG_L(R)_6_3 | 223,224 | *NAC, nucleus accumbens* |
|  |  | BG_L(R)_6_4 | 225,226 | *vmPu, ventromedial putamen* |
|  |  | BG_L(R)_6_5 | 227,228 | *dCa, dorsal caudate* |
|  |  | BG_L(R)_6_6 | 229,230 | *dlPu, dorsolateral putamen* |
|  | Tha (Thalamus) | Tha_L(R)_8_1 | 231,232 | *mPFtha, medial pre-frontal thalamus* |
|  |  | Tha_L(R)_8_2 | 233,234 | *mPMtha, pre-motor thalamus* |
|  |  | Tha_L(R)_8_3 | 235,236 | *Stha, sensory thalamus* |
|  |  | Tha_L(R)_8_4 | 237,238 | *rTtha, rostral temporal thalamus* |
|  |  | Tha_L(R)_8_5 | 239,240 | *PPtha, posterior parietal thalamus* |
|  |  | Tha_L(R)_8_6 | 241,242 | *Otha, occipital thalamus* |
|  |  | Tha_L(R)_8_7 | 243,244 | *cTtha, caudal temporal thalamus* |
|  |  | Tha_L(R)_8_8 | 245,246 | *lPFtha, lateral pre-frontal thalamus* |


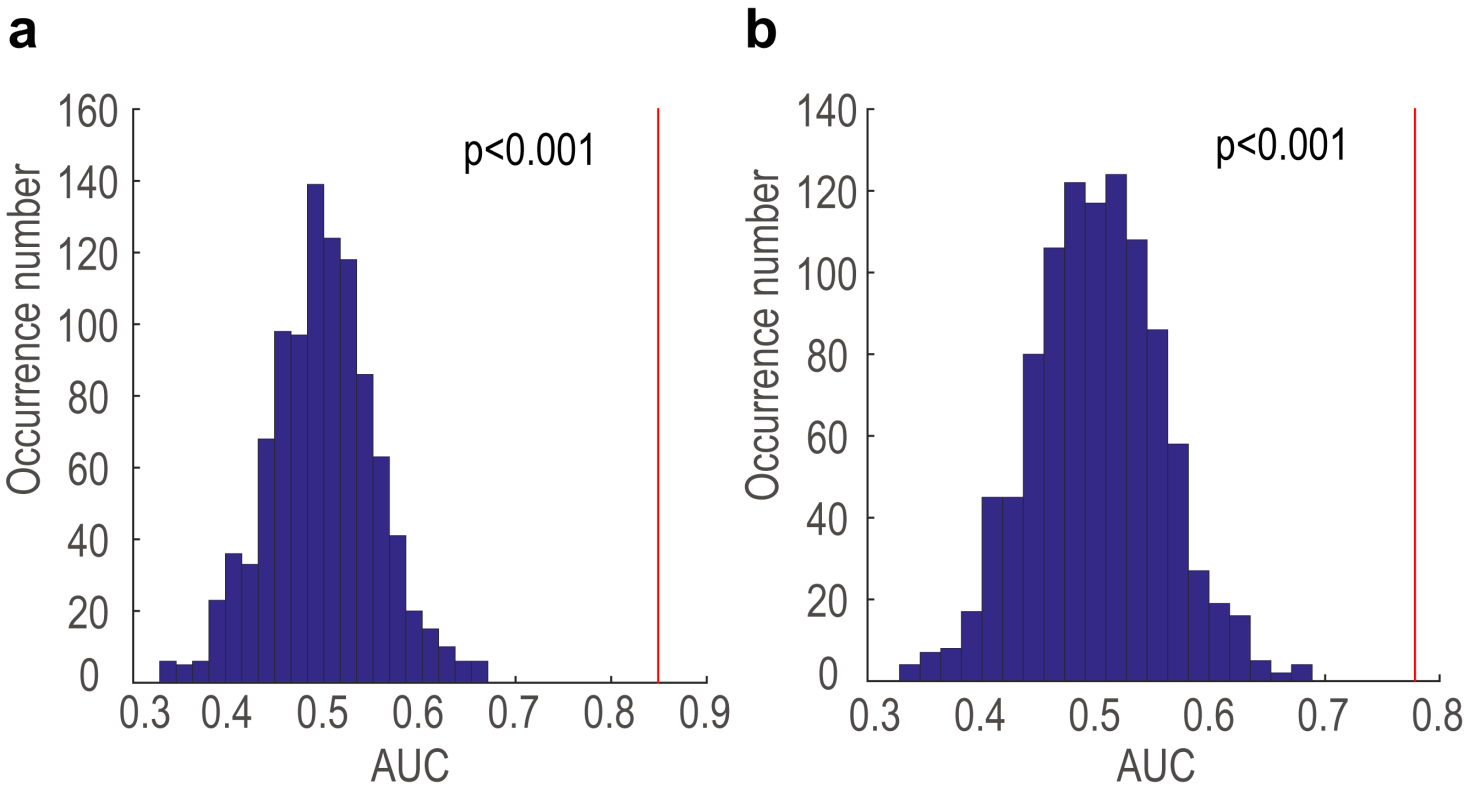


**Figure S1** The distributions of the permutated AUC values without GSR. The distributions of the permutated AUC values of BN-246 atlas (a) and Power-264 atlas (b) without GSR. The red line indicates the values obtained using the real sum predicted label.


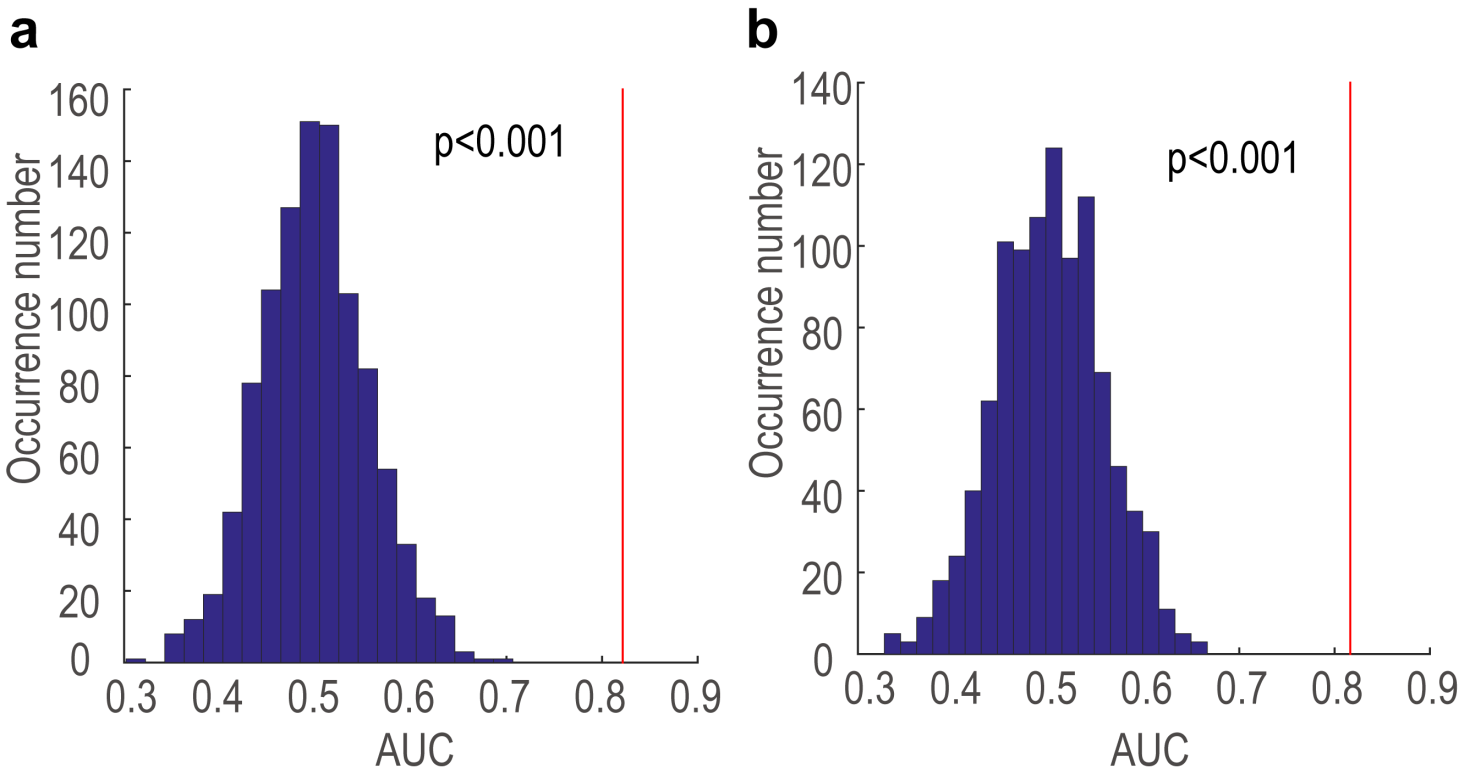


**Figure S2** The distributions of the permutated AUC values with GSR. The distributions of the permutated AUC values of BN-246 atlas (a) and Power-264 atlas (c) with GSR. The red line indicates the values obtained using the real sum predicted label.
